# Understanding the Photocatalytic Activity of La_5_Ti_2_AgS_5_O_7_ and La_5_Ti_2_CuS_5_O_7_ for Green Hydrogen Production: Computational Insights

**DOI:** 10.1021/acsaem.1c03534

**Published:** 2022-01-26

**Authors:** Katarina Brlec, Seán R. Kavanagh, Christopher N. Savory, David O. Scanlon

**Affiliations:** † Department of Chemistry, University College London, 20 Gordon Street, London WC1H 0AJ, U.K.; ‡ Thomas Young Centre, University College London, Gower Street, London WC1E 6BT, U.K.; § Department of Materials, Imperial College London, Exhibition Road, London SW7 2AZ, U.K.

**Keywords:** photocatalytic water splitting, La_5_Ti_2_AgS_5_O_7_ and La_5_Ti_2_CuS_5_O_7_, density functional theory, surfaces, band alignment

## Abstract

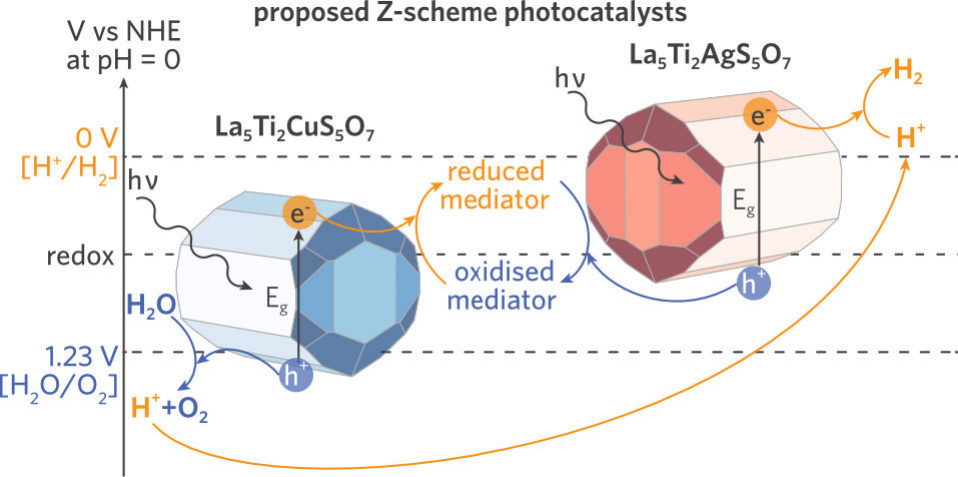

Green production of hydrogen is possible with photocatalytic water splitting, where hydrogen is produced while water is reduced by using energy derived from light. In this study, density functional theory (DFT) is employed to gain insights into the photocatalytic performance of La_5_Ti_2_AgS_5_O_7_ and La_5_Ti_2_CuS_5_O_7_—two emerging candidate materials for water splitting. The electronic structure of both bulk materials was calculated by using hybrid DFT, which indicated the band gaps and charge carrier effective masses are suitable for photocatalytic water splitting. Notably, the unique one-dimensional octahedral TiO_*x*_S_6–*x*_ and tetragonal MS_4_ channels formed provide a structural separation for photoexcited charge carriers which should inhibit charge recombination. Band alignments of surfaces that appear on the Wulff constructions of 12 nonpolar symmetric surface slabs were calculated by using hybrid DFT for each of the materials. All surfaces of La_5_Ti_2_AgS_5_O_7_ have band edge positions suitable for hydrogen evolution; however, the small overpotentials on the largest facets likely decrease the photocatalytic activity. In La_5_Ti_2_CuS_5_O_7_, 72% of the surface area can support oxygen evolution thermodynamically and kinetically. Based on their similar electronic structures, La_5_Ti_2_AgS_5_O_7_ and La_5_Ti_2_CuS_5_O_7_ could be effectively employed in Z-scheme photocatalytic water splitting.

## Introduction

The limited availability of fossil fuel reserves is leading to a global energy shortage. At current consumption rates, petroleum deposits are projected to last until the 2040s while coal deposits should last until the 2200s.^1^ Combined with other renewable energy sources, hydrogen has been identified as a potential energy vector leading the transition to decarbonization of the energy sector. Currently, hydrogen is mass produced by energy-intensive and environmentally unsustainable hydrocarbon reforming that uses the depleting fossil fuels as precursors.^2,3^

Production of hydrogen can be improved by photocatalytic water splitting where water is split into hydrogen and oxygen gas by using the energy derived from light. When hydrogen is burned in the presence of oxygen, water is reformed, effectively keeping the cycle carbon neutral and emissions-free. Since the discovery of the Honda–Fujishima reaction in 1972, TiO_2_ has been extensively studied as a potential photocatalytic water-splitting material.^2,4^ Fujishima and Honda originally reported TiO_2_ as a photoelectrochemical cell electrode, but since it has become one of the most studied photocatalytic systems.^4−6^ The high photocorrosive stability of titania along with its low-cost manufacture and nontoxicity makes it a promising candidate material.^2,5^ However, titania can only absorb light in the ultraviolet range, which only comprises about 5% of the incident solar energy due to its wide band gap (3.2 eV).^7^

To effectively harness the solar energy supply, a good water-splitting material should have a band gap between 1.8 and 2.2 eV.^8,9^ Water splitting is thermodynamically an endothermic reaction, with a positive Gibbs free energy (Δ*G*° = 238 kJ mol^–1^), which leads to a minimum photon energy of 1.23 eV required for thermodynamic water splitting.^10^ However, as more energy is needed to overcome the kinetic overpotential associated with the redox of water, the energy of the photons absorbed should be closer to 2 eV. The material should have a low charge carrier effective mass (below 0.5*m*_e_) in at least one direction to facilitate the mobility of charge carriers to the surface-active sites.^8^

Given these electronic property requirements, understanding the electronic band structure of candidate materials is crucial for the design of photocatalytic water-splitting devices. In titania the O 2p states present at the valence band maximum lie relatively low in energy, thus creating a wider gap to the conduction band minimum composed of Ti 3d states. The relative position of conduction band edges provides a large overpotential for oxygen evolution that ideally should be maintained for maximum photocatalytic performance. The inclusion of chalcogen anions achieves this by introducing higher-energy chalcogen p orbitals that decrease the band gap without altering the conduction band edge.^11^ The quasi-layered Ln_2_Ti_2_S_2_O_5_ (Ln = Y, lanthanides) series exhibit this behavior with S 3p orbitals forming on the top of the valence band and O 2p states at a lower energy level.^12,13^ This makes these systems candidates for both reduction and oxidation of water with good band edge alignment.^12^ Additionally, the hybridization of O 2p and S 3p states in mixed anions further stabilizes the surface S^2–^ to self-oxidation and photocorrosion in aqueous solutions.^13^

**Figure 1:**
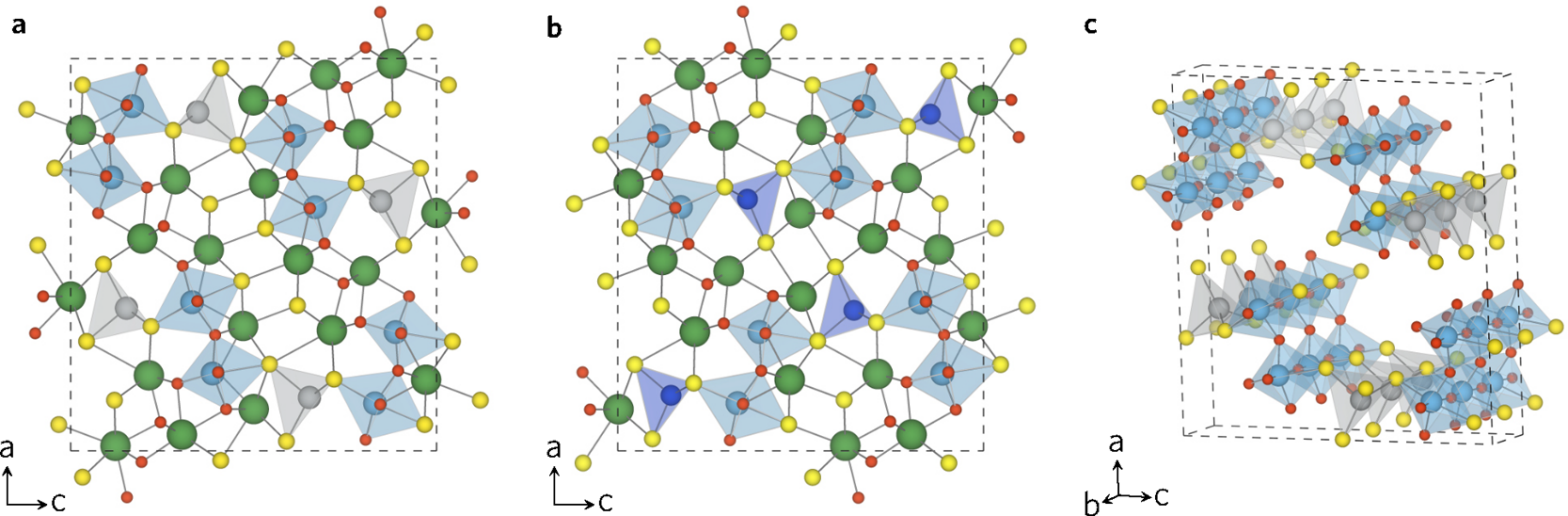
Crystal structures of (a) LTA and (b) LTC as seen down the *b*-axis. The TiO_*x*_S_6–*x*_ octahedra (light blue) and the rock-salt-like AgS_4_ (silver) and CuS_4_ (dark blue) are shown while the fluorite-type La polyhedra are omitted for clarity. (c) One-dimensional tetrahedral and octahedral chains in a 1 × 3 × 1 LTA supercell. La = green, Ti = blue, S = yellow, O = red, Ag = silver, and Cu = dark blue.

Inspired by the Ln_2_Ti_2_S_2_O_5_ series, Meignen et al. posed that introduction of a late transition metal such as Cu or Ag could enhance the segregation of layers.^14^ Thus, La_5_Ti_2_AgS_5_O_7_ (LTA) and La_5_Ti_2_CuS_5_O_7_ (LTC) were first synthesized by Meignen et al. in 2004 and have since been shown to exhibit photocatalytic activity for both reduction and oxidation of water in the presence of Pt and IrO_2_ cocatalysts.^14−16^ The systems are reported to have band gaps of 2.17 eV (LTA) and 1.91 and 2.02 eV(LTC).^14,17^ Nevertheless, the initial hypothesis of Cu/Ag promoting layer separation was incorrect, with only fragments of layers observed in the [La_5_Ti_2_MS_5_O_7_]_4_ (M = Ag, Cu) orthorhombic (*Pnma*, No. 62) unit cells (see Figure 1).

The recombination of charge carriers is expected to be limited due to the presence of the one-dimensional tetrahedral MS_4_ (M = Ag, Cu) and octahedral TiO_*x*_S_6–*x*_ chains in the crystal structure.^17^ This is based on the idea that a layered structure would increase the separation of photogenerated charge carriers by effectively trapping them individually on different chemical motifs in the structure, separated by insulating layers. For example, in Sr_2_Cu_2_ZnO_2_S_2_ the electron transport occurs via the (ZnO_2_)^−^ layer, the holes are transported via the (Cu_2_S_2_)^2–^ layer, and the Sr^2+^ layer is found between the former two. In the case of LTA and LTC, the octahedral and tetrahedral chains in the structure are expected to enable the charge carriers to reach the surface sited efficiently without significant recombination effects.^18^ Recently, analysis of needle-shaped LTC single crystals has proven the existence of one-dimensional anisotropic electronic states along the *b*-axis.^19^

LTA and LTC have been researched as potential water-splitting materials with the focus on various dopant–cocatalyst combinations as well as the materials’ unique structure.^15,17,19−23^ Solid solutions of LTA and LTC have been computationally studied, but no comprehensive study of the surface energetics has been performed so far.^24^ This study aims to present a high-level computational overview of the bulk and surface electronic structure, with an emphasis on combining the surface energetics and band alignment to explain the experimentally observed trends in photocatalytic activity.

## Computational Methodology

All calculations were performed by using Vienna ab initio Simulation Package (VASP), based on periodic density functional theory (DFT).^25−27^ VASP recommended projector augmented wave (PAW) potentials were used in the calculations. To choose the optimal *k*-point meshes and kinetic energy cutoff, the energy was converged to <1 meV atom^–1^ by using the generalized gradient approximation functional Perdew–Burke–Ernzerhof revised for solids (PBEsol).^28^ The plane wave energy cutoffs of 450 and 500 eV were used for LTA and LTC, respectively. The Brillouin zone for bulk materials was sampled by 1 × 5 × 1 Γ-centered *k*-point mesh for geometry relaxations and electronic band structures. The tetrahedron method with Blöchl corrections and a denser 2 × 10 × 2 Γ-centered mesh was employed to calculate the density of states due to a higher sensitivity of calculations.

The geometric structures were obtained from the Inorganic Crystal Structure Database (ICSD) and relaxed into the ground state by using the PBEsol functional which has been shown to accurately calculate lattice parameters for solid state semiconductors.^14^ The structures were relaxed until the force on any of the atoms did not exceed 0.01 eV Å^–1^. For calculation of bulk electronic properties, including accurate assessments of the band gaps and alignments, exact exchange Hartree–Fock and DFT methods were implemented with the use of the range-separated hybrid functional Heyd–Scuseria–Ernzerhof (HSE06) and PBE0 functional.^29−31^

The surfaxe package was used to cleave the surface slabs.^32^ All zero-dipole symmetric slabs up to a maximum Miller index of two were considered in this study. The (001) surface was added, and the (1–12) and (−211) surfaces replaced the (112) and (211) surfaces as they were observed on selected area electron diffraction (SAED) patterns by Iwase et al.^19,20^ The pairs of positive and negative index surfaces were identical, but the negative index slabs were included for consistency with experimentally reported nomenclature. Slab calculations used the same energy cut off as bulk, with *k*-point meshes derived from the bulk, keeping the same reciprocal space spacing. Convergence testing was done for slab and vacuum thickness with respect to surface energy with convergence criterion of 0.02 J m^–2^. If a Miller index had multiple valid terminations, the one with lowest energy and flat electrostatic potential in a vacuum was chosen for further investigation.

The positions of all atoms in slabs were relaxed with PBEsol with constraints on cell shape and volume by using the same convergence criteria as for the bulk. Single-shot PBEsol calculations were done on the relaxed surface slabs to obtain accurate energies. Based on surface energies of the slabs and the bulk crystal structure, Wulff constructions were drawn for the systems by using the Pymatgen Analysis module.^33^ Band alignment of LTA and LTC surface slabs was also calculated by aligning the vacuum potential from slabs to the bulk by using core levels from HSE06 single-shot calculations on PBEsol relaxed structures.^34^

## Results and Discussion

### Bulk Calculations

The relaxed crystal structures of LTA and LTC viewed down the *b*-axis can be seen in Figure 1. The compounds have isostructural centrosymmetric orthorhombic crystal structures with offset origins. As described by Meignen et al., the complex quinary crystal structure can be broken down into three components: rock-salt-like MS_4_ tetrahedra, double chains of TiO_*x*_S_6–*x*_ (*x* = 4, 5) corner-shared octahedra, and various fluorite-type LaO_*y*_S_*z*_ segments. The tetrahedral and octahedral units formed one-dimensional chains in the *b*-direction, highlighted in Figure 1c.

Geometric relaxation of LTA and LTC was performed with PBEsol due to the size and complexity of the 80-atom quinary system. The lattice parameters of both systems (see Table 1) have decreased by <2% during the relaxation relative to the experimental structures, which was in line with expected PBEsol performance. A loss of symmetry below the threshold of 0.005 Å was observed during the relaxation of LTC when the symmetrization of charge density was not directly imposed in the calculation. The resulting *P*1 structure was 0.001 meV atom^–1^ lower in energy compared to the *Pnma* structure that was relaxed with the symmetry precision set at 1 × 10^–5^ Å. The difference in fractional coordinates of atoms of the *P*1 and *Pnma* structures were on the order of 1 × 10^–5^, well below the DFT error margins (see the Supporting Information for structure files). Going forward, the *P*1 structure was used for LTC as the lower energy structure.

**Table 1:** Lattice Parameters for LTA and LTC as Calculated with PBEsol and the Percentage Deviation from Experimental Values^14^

	LTA	LTC
*a*/Å	19.38 (−1%)	19.26 (−0.8%)
*b*/Å	3.93 (−1.6%)	3.93 (−1.4%)
*c*/Å	18.10 (−1%)	17.97 (−0.8%)
α/deg	90	90
β/deg	90	90
γ/deg	90	90

The relaxation had no impact on the coordination of the atoms in the unit cell. The coordination environment was determined by examining the structures using the longest bond length for each bond as reported by Meignen et al.^14^ The descriptions of bonding environments held true for both LTA and LTC, as they are isostructural. La exhibited three different environments, as seven-coordinate LaO_3_S_4_, eight-coordinate LaO_3_S_5_, and nine-coordinate LaO_4_S_5_. All Ti were in octahedral environments as either TiO_5_S or TiO_4_S_2_, while Ag or Cu appeared in the MS_4_ tetrahedra. All O atoms were four-coordinate whereas S were either five- or six-coordinate.

The LTA and LTC electronic band structure and density of states were evaluated by using three functionals: PBEsol, PBE0, and HSE06. Spin–orbit coupling (SOC) was included at the PBEsol level to assess the extent of effect SOC has on the electronic structure. Band gaps for both systems are reported in Table 2. The LTA band gap was direct; however, both direct and indirect gaps were observed for LTC. While the fundamental band gap of LTC was indirect, direct transitions between valence band maximum and conduction band minimum were available very close in energy. The PBE0 band gaps included were obtained from a density of states calculation as the lowest energy direct transition between valence band maximum (VBM) and conduction band minimum (CBM).

**Table 2:** Band Gap Energies for Bulk LTA and LTC Calculated with PBEsol, PBEsol with Spin–Orbit Coupling (SOC), HSE06, and PBE0 Functionals Compared with Experimental Work and Band Gaps Calculated with PBE^14,17,24^ a

functional	LTA *E*_g_/eV	LTC *E*_g_/eV
PBEsol	1.51	1.261, 1.259^†^
PBEsol+SOC	1.49	1.25
HSE06	2.65	2.415, 2.413^†^
PBE0	3.38^‡^	3.15^‡^
experimental	2.17^14^	2.02,^14^ 1.91^17^
PBE^24^	1.55	1.29

a Experimental band gaps were obtained by Kubelka–Munk UV–vis diffuse reflectance spectroscopy on single crystals. Indirect band gaps denoted with †. Band gaps calculated from density of states denoted with ‡.

Following the experimentally determined trend, LTA had a consistently larger band gap compared to LTC. The PBEsol band gaps of 1.51 eV (LTA) and 1.26 eV (LTC) agreed well with the literature band gaps of 1.55 and 1.29 eV calculated with PBE.^24^ Both PBEsol and PBEsol+SOC calculated band gaps were narrower compared to the experimental values due to the systematic underestimation of band gaps for semiconductors by the functional. The inclusion of SOC decreased the band gap by about 1%, to 1.49 and 1.25 eV for LTA and LTC, respectively, so the SOC effects were not considered at higher levels of theory. As PBE0 is best suited for wide gap semiconductors and insulators, the functional overestimated the band gap by over 1 eV compared to experimental value. HSE06 best replicated the literature values with band gaps of 2.65 eV for LTA and 2.415 eV for LTC. The band gap in LTA was direct, with VBM and CBM both occurring at Γ. The indirect band gap observed in LTC had placed the CBM between Γ and X at [0.00, 0.14, 0.00]. The CBM lay 0.002 eV lower in energy than Γ.

**Figure 2:**
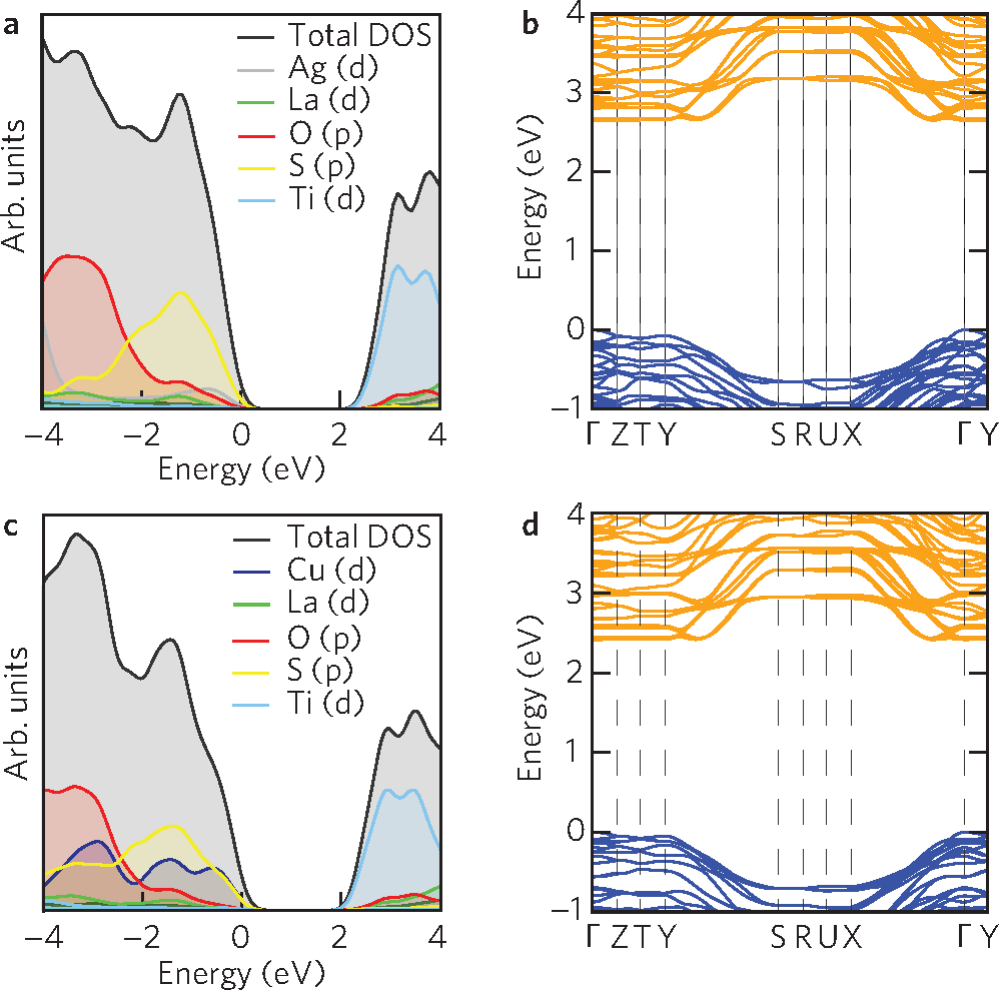
LTA (a) density of states and (b) band structure. LTC (c) density of states and (d) band structure as calculated with HSE06 functional on a PBEsol relaxed structure. Plotted using sumo.^35^

The electronic band structures of LTA (Figure 2b) and LTC (Figure 2d) exhibit similar features, which is expected due to the isostructural lattice. The band topology around Γ, where direct band gaps exist for both systems, was flat with the exception of Γ → X direction which corresponded to the *b*-direction in real space. The curvature of the bands is correlated to the unique one-dimensional TiO_*x*_S_6–*x*_ and MS_4_ chains seen in Figure 1c.

The effective masses were calculated by using parabolic fitting of the band edges (see Table 3). The curvature in the valence band around Γ led to relatively low hole effective masses between Γ and *X*, *Y*, or *Z*. The hole effective mass was the lowest in the Γ → *X* direction at 0.392*m*_e_ in LTA and 0.410*m*_e_ in LTC. The lowest effective electron mass in LTC (CBM → X) was 0.47*m*_e_, lower than the comparable effective mass in LTA (1.14*m*_e_ Γ → *X*). This was a consequence of the indirect band gap in LTC as the CBM fell just short of Γ and thus allowed for the disperse part of the band to become available for charge carrier movement. As mobility and charge carrier effective mass are inversely proportional, the mobility was expected to be the highest between Γ and *X* in both systems. High predicted mobility in LTC is in line with experimental results, corroborating the ease of charge carrier movement along the *b*-axis. In LTA the electron mobility was predicted to be lower than in LTC with effective electron mass of over 0.5*m*_e_ in the conduction band which could lead to lower photocatalytic activity. On the basis of effective charge carrier masses, it is expected that holes will diffuse to active sites on the surface of photocatalysts effectively in both LTA and LTC, whereas electrons will diffuse more effectively in LTC.

**Table 3:** LTA and LTC Effective Charge Carrier Masses Calculated by Parabolic Fitting of the Band Edges

	effective hole mass/*m*_e_	effective electron mass/*m*_e_
LTC	0.39 Γ → *X*	1.14 Γ → *X*
	1.34 Γ → *Y*	7.13 Γ → *Y*
	1.22 Γ → *Z*	>10 Γ → *Z*
LTC	0.41 Γ → *X*	0.47 CBM → *X*
	1.81 Γ → *Y*	1.85 CBM → Γ
	1.79 Γ → *Z*	

Like the band structures, the density of states (DOS) of LTA (Figure 2a) and LTC (Figure 2c) were similar. In terms of partial DOS, the conduction band composition was similar to the presence of Ti(d), O(p), and La(d) orbitals. In LTA, the DOS was composed of S(p), O(p), and Ag(d) orbitals between the VBM and 2 eV below the VBM. In LTC, S(p), Cu(d), and O(p) were present over the same energy range, with Cu(d) proportion increasing significantly.

The analysis of composition of band edges corroborated the findings from previous qualitative studies. As expected, the composition of CBM was similar between the systems, whereas the composition of VBM differs. The CBM in both materials was composed mainly of Ti(d) orbitals, 87% in LTA and 86% in LTC. O(p) orbitals accounted for 9% and La(d) for 5% of CBM in both systems. In the LTA VBM S(p) (66%) and Ag(d) (20%) orbitals dominated, with 4% Ti(d) and O(p) presence each. Similarly, the LTC VBM was also predominantly S(p) and Cu(d) in character, accounting for 46% and 40%, respectively, with the remaining 14% split among Ti(d), O(p), and Cu(p) orbitals. This quantified the literature observations that Ag 4d orbitals were too low in energy to sufficiently hybridize with S 3p orbitals in LTA while LTC Cu 3d and S 3p hybridize successfully.

**Figure 3:**
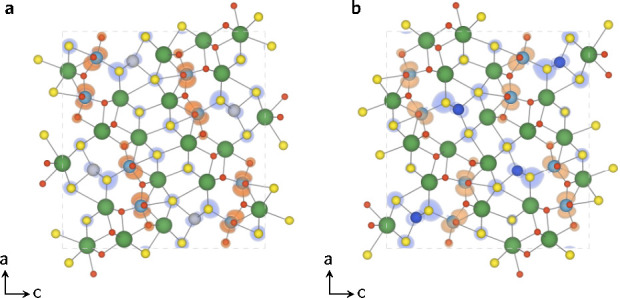
Band edge charge densities for (a) LTA and (b) LTC visualized with VESTA.^36^ Valence band maxima (blue) are centered around the MS_4_ (M = Ag, Cu) tetrahedra in LTA and LTC. Corner-sharing between MS_4_ and TiO_4_S_2_ leads to some charge density observed on the S–Ti–S axis, mostly centered around S atoms. The greatest valence band maxima charge density is on the S atom shared between MS_4_ and TiO_4_S_2_. Conduction band minima (orange) are centered on the Ti(d) and O(p) orbitals in the equatorial TiO_4_ of the TiO_*x*_S_6–*x*_. La = green, Ti = light blue, S = yellow, O = red, Ag = silver, and Cu = dark blue.

The band edges can be visualized by mapping the partial charge density at a specific *k*-point and electronic band to the structure of the material. Figure 3 shows the (a) LTC and (b) LTC VBM in blue and CBM in orange. As expected, the CBM is centered around the TiO_*x*_S_6–*x*_ units, in particular on the equatorial TiO_4_. The VBM in both materials is centered around the MS_4_ (M = Ag, Cu) tetrahedra; however, because of corner sharing of the MS_4_ and TiO_4_S_2_ units charge spills out via the S–Ti–S and Ti–O–Ti axes onto the neighboring TiO_4_S_2_. This accounts for 14% of the charge density, most of which is centered around Ti(p) and O(p) orbitals. One-dimensionality in charge carrier transport was established in many experimental studies; however, Suzuki et al. showed a similar overspill of charge density onto TiO_*x*_S_6–*x*_ in their highest occupied molecular orbital/lowest unoccupied molecular orbital diagram.^17−20^ The diffuseness of VBM charge density could explain the performance of LTA and LTC as overall photocatalysts for water splitting as charge carriers are not as confined to their one-dimensional chains in the valence band. The CBM is firmly located on the equatorial plane of TiO_*x*_S_6–*x*_, so easier spatial movement of photoelectrons could be expected.

### Slab Construction and Relaxation

All surface slabs considered in this study were symmetric and nonpolar. The former is a consequence of the slab model assumption that the surfaces of the slab are the same, whereas the latter ensures a low surface energy. If a surface of an ionic crystal is charged, the existence of dipole moment perpendicular to the surface will likely induce a polarizing electric field which consequently results in a large surface energy.^37^ Polar surfaces are also more likely to undergo reconstruction which would add an additional degree of complexity to already complex systems. For each of the bulk crystals, 142 surface terminations were available in total; however, only 15 terminations across all Miller indices satisfied the two requirements. No restrictions were placed on the number or character of bonds broken in each cut as none of the bonds in the systems were identified as strong covalent bonds.

Some exceptions were made to the symmetry requirement as slabs inherit symmetry operations from the bulk structure. All LTC slabs were only symmetric up to the threshold of 0.005 Å because of the aforementioned loss of symmetry during the relaxation of the bulk. Still, the symmetry tolerance was greater than the default symmetry tolerance of Pymatgen modules that deal with slab creation and analysis (0.1 Å). The (001) surface slabs were noncentrosymmetric with horizontal mirror plane symmetry.

The surface energy was converged with respect to thickness of slabs and vacuum. This was done to ensure the center of the slab is as bulk-like as possible and that the surfaces do not interact with each other. The vacuum thickness was chosen to be at minimum 30 Å while the slab thickness varied between the Miller indices, but at least two 80-atom layers were included in each slab. All slabs are included in the Supporting Information. For Miller indices with multiple zero dipole symmetric terminations, the lowest energy surface slab with flat vacuum potential was chosen for further investigation.

The constructed slabs have bulk-like surfaces with high excess energy due to the formed dangling bonds so the surfaces must be relaxed. All atoms were allowed to relax position, while simulation cell shape and volume were kept consistent to allow comparison with the bulk unit cell. Because of the large size of slabs with hundreds of atoms included the PBEsol functional was used to relax the slabs. The behavior of isostructural LTA and LTC slabs of the same Miller index during the relaxation was similar. In general, the atoms on the surface displaced by over 0.2 Å, with only minor changes to the position of atoms in the center of the slab. Some changes in coordination environment resulting from a relaxation were observed on the surface La atoms, most commonly related to movement of S and O atoms around Ti-centered octahedra or MS_4_ tetrahedra. In all but (001) and (100) slabs, the cleavage cut through the M–S bond in MS_4_ so the MS_3_ formed flattened out to a trigonal-planar structure during the relaxation. Two M–S bonds were cleaved in surface formation in (102), (122), (201), (−211), and (212), where during the relaxation the S–M–S angles widened to up to 169°.

More complexity in cleavage and relaxation was observed on the TiO_*x*_S_6–*x*_ octahedra, in part due to the unusually long Ti–S bond and multiple Ti coordination environments but also due to a greater number of possible bond-breaking combinations. On most surfaces, the Ti atoms with dangling bonds remain undercoordinated after the relaxation with no changes to the coordination environment observed. On the (001) surface only one Ti–S bond was broken in what should have been a TiO_4_S_2_ unit. In (010) and (122) all four TiO_*x*_S_6–*x*_ octahedra were cleaved along the axial direction so all four had one equatorial Ti–O bond missing. The (011) surface opened up four Ti-centered octahedra, with two TiO_4_S_2_ missing one Ti–S and one equatorial Ti–O bond each and two TiO_5_S missing one equatorial Ti–O bond. While no Ti-based bonds were broken when the (102) surface was created, the surfaces of (100) and (101) only had one Ti–O dangling bond—in equatorial and axial positions, respectively. Three different types of TiO_*x*_S_6–*x*_ cleavage occur on the (110) surface where equatorial Ti–O bonds were left dangling; two TiO_4_S_2_ and one TiO_5_S were cut along the axis to cleave one bond each, and another TiO_5_S lost two bonds. Both surface TiO_4_S_2_ on the (201) had a dangling Ti–O bond, while one of the two Ti also had a broken Ti–S bond, leaving it four-coordinate.

**Figure 4:**
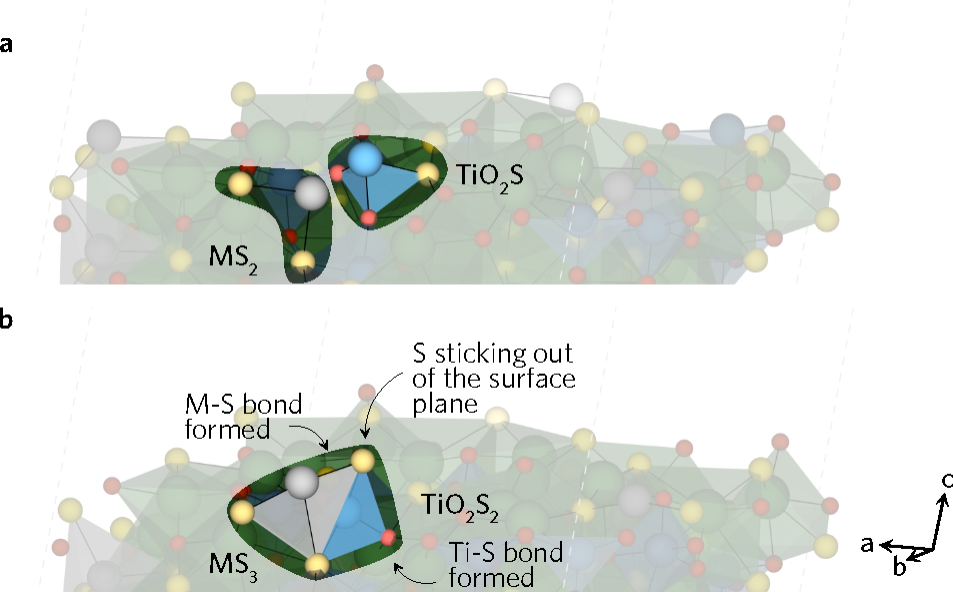
(1–12) LTA (a) unrelaxed and (b) relaxed surface.

**Figure 5:**
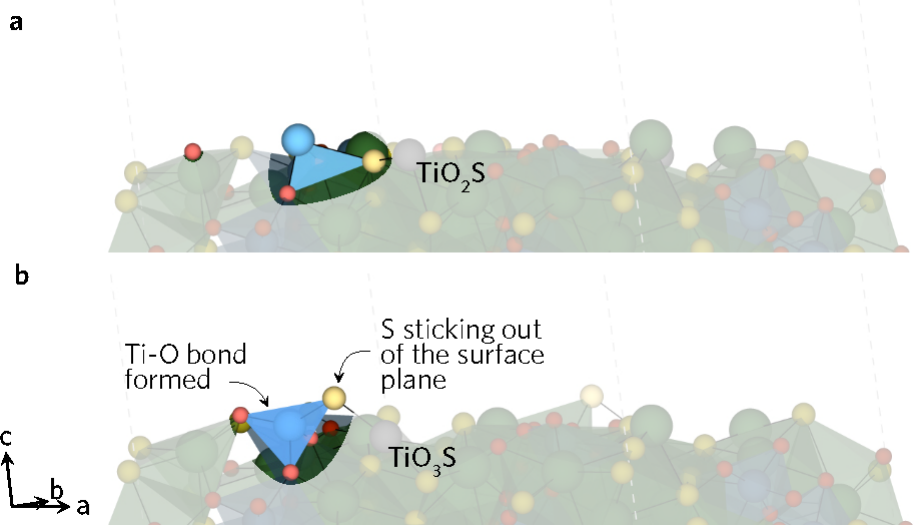
(−211) LTA (a) unrelaxed and (b) relaxed surface.

Where the surface creation left Ti in a three-coordinate state, regardless of which bonds remained intact, the surface reconstructed. Generally, Ti-based reconstructions seem to correspond to Ti moving down from the exposed surface into the material, while pushing the bonded S up toward the surface. In addition to dangling Ti–S and equatorial Ti–O bonds from TiO_4_S_2_ and TiO_5_S on the (1–12) surface that did not change coordination environments during the relaxation, the three-coordinate Ti and its neighboring two-coordinate M atom underwent a reconstruction (see Figure 4). This resulted in a tetragonal TiO_2_S_2_ and a trigonal-planar MS_3_ positioned perpendicular to the surface plane. The M–S bond distance on another MS_3_ unit increased to over 3.5 Å, effectively breaking the bond. The motion of Ti and M atoms in the *c*-direction effectively resulted in switched positions with Ti pushed down into the bulk and M moved onto the surface. Similarly, on the (−211) surface the TiO_3_S unit highlighted in Figure 5 that was missing two equatorial and one axial Ti–O bond formed a Ti–O bond with an O on the neighboring La motif to remove on set of Ti-based dangling bonds. The other undercoordinated TiO_*x*_S_6–*x*_ remain in their coordination environments during the relaxation.

Two different (212) surfaces were cleaved from LTA and LTC. The LTC (212) surface cleaved three Cu–S bonds, an equatorial Ti–O bond in a TiO_4_S_2_ and TiO_5_S, and two equatorial Ti–O bonds in another two TiO_4_S_2_ and TiO_5_S. The latter two units also had one of the axial bonds cleaved, Ti–S and Ti–O, respectively. The surface did not undergo a reconstruction; however, during the relaxation a Cu–S bond in one of the surface CuS_4_ increased so the motif flattened to the trigonal-planar CuS_3_ configuration.

**Figure 6:**
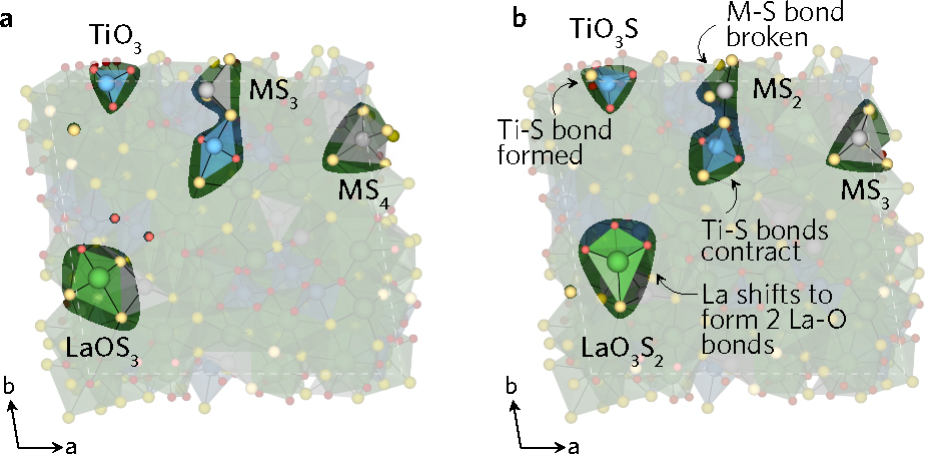
(212) LTA (a) unrelaxed and (b) relaxed surface.

More Ag–S, Ti–O, and Ti–S bonds were broken in the formation of the LTA surface compared to LTC. Three Ti units that did not reconstruct had one equatorial Ti–O dangling bond, with one of them also missing a Ti–S bond. The S–Ti–S bond angle in TiO_2_S_2_ highlighted in Figure 6 decreased from 180° to 118°, which resulted in neighboring AgS_3_ displacing closer to the surface, breaking one of the Ag–S bonds and leaving the Ag two-coordinate. The most notable observed reconstruction occurred on the other highlighted TiO_3_ where a neighboring S atom displaced to form a TiO_3_S tetrahedron that stuck out of the surface. This caused the bond distance between the displaced S and the Ag it was bonded to increase to over 3 Å, effectively breaking the Ag–S bond. Lastly, the highlighted La atom displaced, breaking a La–S bond but also creating two new stronger La–O bonds.

### Surface Energies and Wulff Constructions

PBEsol single-shot calculations using Gaussian *k*-point smearing were done on the relaxed systems to obtain accurate slab total energies. Surface energies were then calculated by using the following equation:
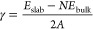
1where *E*_slab_ is the total energy of the slab, *N* is the number of atoms in the slab, *E*_bulk_ is the bulk energy per atom, and *A* is the base area of the slab. Surface energies are sensitive to the bulk energy used in the calculation, as there is a systematic discrepancy in the *k*-space sampling between slab and bulk unit cells. While the use of bulk energy derived from the linear fit of a number of slab total energies is generally preferred, this approach was infeasible with LTA and LTC due to the size of some of the slabs as the number of atoms increased in lots of 80, 160, or even 320 atoms for each added layer.^38^ Bulk energy used in calculation of surface energy was derived from the total bulk energy of the conventional unit cell, calculated with same level of theory and smearing parameters as the slab total energy.

Comparing the energies between the Miller indices of the two materials, surface energies were similar and follow the same ordering. Lower index surfaces generally had lower surface energy as a result of fewer strong (Ti–O axial and equatorial, M–S) bonds broken compared to their higher index counterparts. The relaxation of the slab also played a role in the energetics on the surface. While more bonds were broken on the (1–12) and (−211) surfaces than on the (011), the reconstructions on the higher index surfaces decreased surface energies. The difference in energies of the (212) surfaces between LTA and LTC was relatively large at 0.25 J m^–2^, likely originating from different reconstructions taking place on the surface.

Wulff constructions of equilibrium forms of LTA and LTC single crystals were determined based on the surface energies, seen in Table 4, and bulk structures. The proportions and shapes of facets on the crystals differ slightly between LTA and LTC. Neither system displays the extent of anisotropy described by Iwase et al., likely due to the experimental conditions favoring elongation of crystals.^19,20^ The surface energy anisotropy, based on the coefficient of variation from weighted surface energy, is 0.098 for LTA and 0.111 for LTC, making LTC ever so slightly more anisotropic. Both materials are predicted to be more anisotropic than 88% of the elemental crystals.^39^

**Table 4:** LTA and LTC Surface Energies as Calculated with PBEsol on a PBEsol Relaxed Slab

surface	LTA γ/J m^–2^	LTC γ/J m^–2^
(001)	1.244	1.207
(010)	0.931	0.944
(011)	1.168	1.094
(100)	0.801	0.790
(101)	0.817	0.859
(102)	0.866	0.908
(110)	0.953	1.004
(1–12)	1.070	1.083
(122)	1.128	1.150
(201)	1.496	1.570
(−211)	1.064	1.096
(212)	1.193	1.372

**Figure 7:**
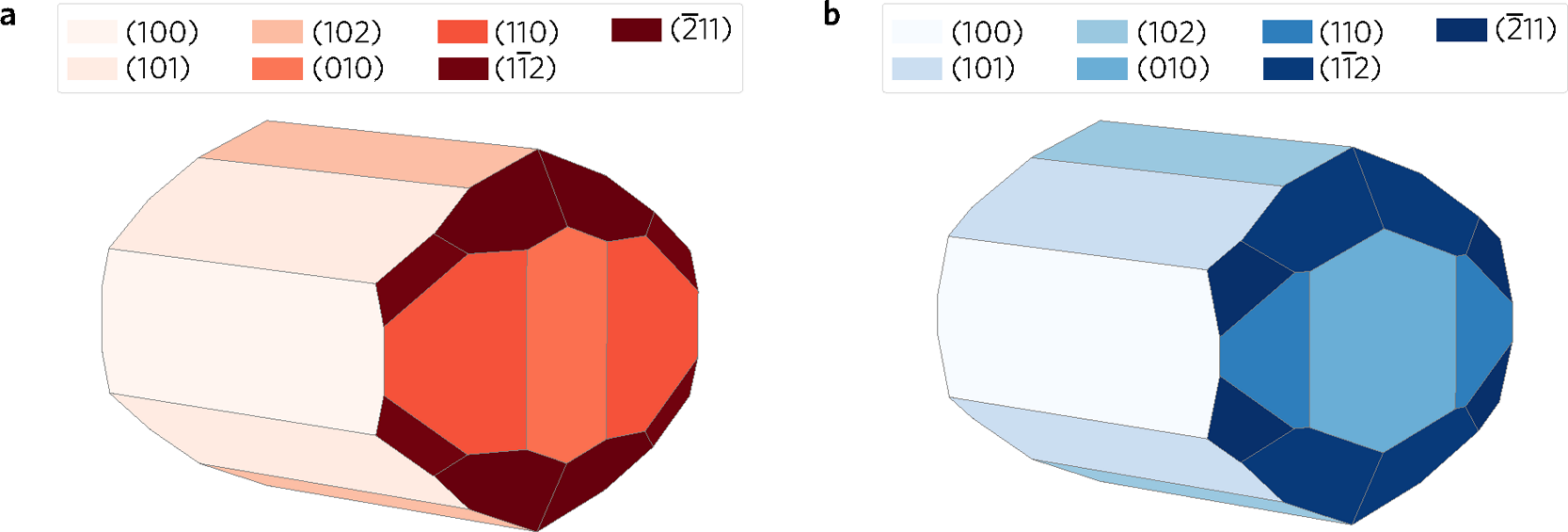
Wulff constructions of (a) LTA and (b) LTC. The surfaces are color-coded where lighter shades represent lower surface energy and darker shades represent higher surface energy.

LTA Wulff shape seen in Figure 7a realized 7 of the 12 investigated Miller indices. Surface energies of the omitted five were over 10% greater compared to the remaining surfaces. Lower energy was not always directly correlated to a larger percentage of the total area observed on the Wulff shape for the facets as this additionally depends on competition with neighboring nonorthogonal facets. In fact, the largest facet was not the lowest energy one—the (101) facet covered 29% of the total area. The remaining facets in descending order by percentage of total area they cover were (100) and (102) with 18%, (110) with 14%, (1–12) with 10%, (010) with 7%, and (−211) with 4%. Similarly to (100) and (101), (1–12) and (−211) combined covered a much greater surface area compared to (010) even though the surface energies were greater by 0.139 and 0.133 J m^–2^, respectively.

The LTC Wulff shape in Figure 7b was unsurprisingly similar to the LTA Wulff shape. Seven of the studied surfaces appear on the construction—again, surface energies of the missing surfaces were over 10% greater than the highest energy surface present on the Wulff construction. The (−211) surface energy was 0.02 J m^–2^ greater than the (011) surface energy, but as the combination of (1–12) and (−211) surfaces led to an overall lower total surface energy, the higher energy surface was realized regardless. As in LTA, the (101) facet covering the greatest total area was not the one with the lowest energy. The order of surfaces by the percentage area covered was different though—the top three surfaces were still (101), (100), and (102), covering 26%, 20%, and 18% of total surface area, respectively. These are followed by (1–12) covering 14%, (010) with 11%, (110) with 6%, and last (−211) with 5%. The correlation of lower energy to higher surface area was again disputed here, as the surface energy of (1–12) was greater than of all but the (−211) facet.

The shape of the LTC Wulff construction can be compared to molten-salt flux grown crystallites reported by Iwase et al.^19,20^ While the (010) end of the Wulff shape resembled the blunt ends of crystallites well, the length of the predicted columnar particle was very different. This was most likely due to the flux-mediated method used in the experimental work favoring elongation of crystals in the *b*-direction, something that cannot be accounted for during the construction of the Wulff shape. The selected-area electron diffraction (SAED) patterns from studies by Iwase et al. correlate well with the results from this study. Only (001) and (201) which appeared on one of the experimental patterns were absent from theoretical Wulff shape proposed here—both were predicted to have significantly higher surface energy compared to the other facets on the Wulff shape.

The calculation of the surface energies assumes a dry environment with no water medium present, which is obviously not the case in real-world water-splitting photocatalysis. The surface energy equation (eq 1) can be extended to include solvation and adsorption effects; however, the computational cost required to compute those is beyond the scope of this investigation.^40^ Nevertheless, some conclusions may be drawn from the literature. In general, a decrease in surface energy is observed with an increase in hydration, with higher energy surfaces experiencing a greater stabilizing effect upon adsorption.^41,42^ For facets that are close in energy the relative ordering may change, so in the case of LTA some reordering of the (100)–(101) and (1–12)–(−211) surface pairs may be expected as their respective surface energies are similar. Lastly, as the range of surface energies is likely to decrease, the Wulff shape is expected to become more isotropic.

### Band Alignment

To assess whether the material can reduce and oxidize water, the material’s band edges must straddle the redox potentials. In other words, the VBM must lie at a potential lower than −5.76 eV whereas the CBM must lie higher than −4.44 eV at pH = 7. To obtain the band alignment, the energy of the system in relation to the vacuum needs to be calculated to get the values of ionization potential (IP) and electron affinity (EA). The method derived by Wei and Zunger was used to obtain these values.^43^ The IP relates the vacuum level to the VBM of bulk, so this can be expanded into the following equations by using bulk and slab core levels:
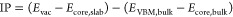
2
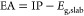
3where *E*_vac_ is the vacuum level, *E*_core,slab_ is the core level of an atom in the slab, *E*_VBM,bulk_ is the bulk VBM, *E*_core,bulk_ is the core level of an atom in the bulk, and *E*_g,slab_ is the band gap of the slab. To ensure compatibility between bulk and different slabs, the O atom in core level alignment was always chosen from the same environment; this was the mid-slab O atom with three bonded La atoms and one Ti atom. The vacuum level was obtained from the plateau in the electrostatic potential of the slabs in the vacuum region. For the band alignment of bulk, the (010) slab was chosen to align the unrelaxed core state with the bulk core state to get the bulk IP from eq 2, and bulk band gaps were used to obtain bulk EA from eq 3.

**Figure 8:**
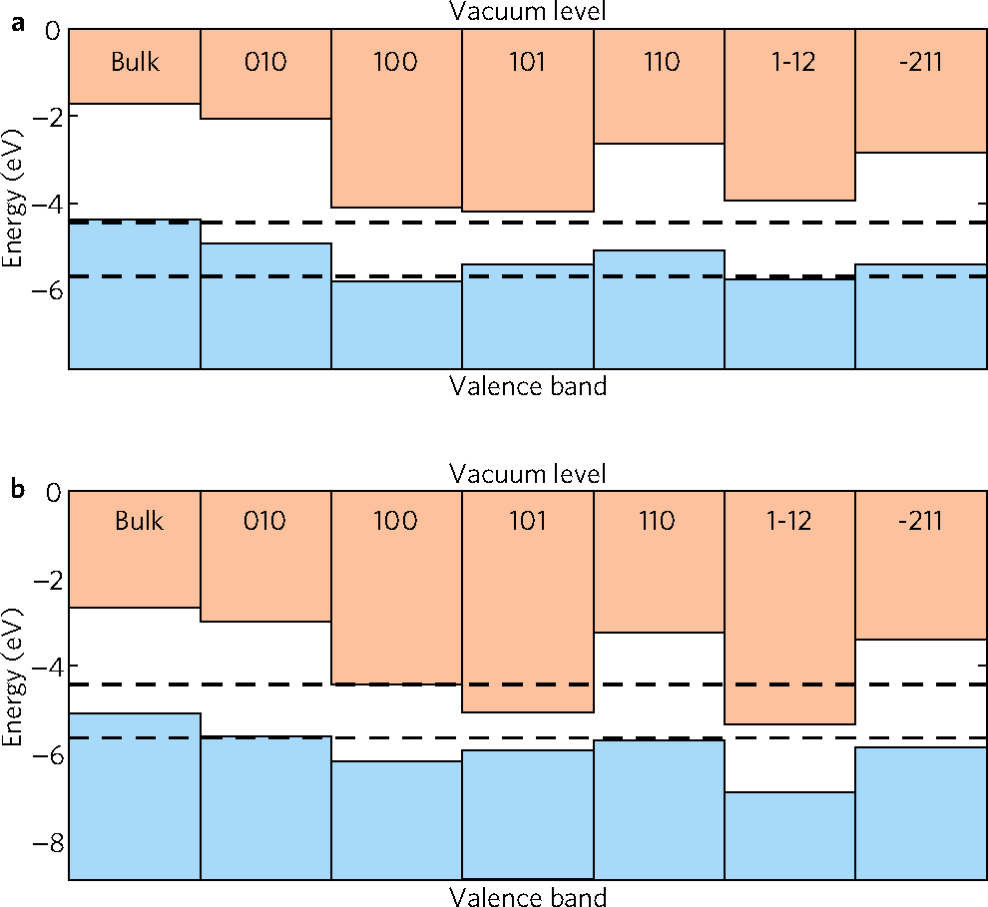
Band alignment of (a) LTA and (b) LTC as calculated with HSE06 on a PBEsol relaxed structure. The dashed lines represent the redox potentials of water at pH = 0.

As can be seen in Figure 8a, all studied LTA surfaces satisfied the thermodynamic requirement for hydrogen evolution photocatalysis. The (010), (110), (1–12), and (−211) surfaces accounting for 34% of the total Wulff area showed excellent overpotential for hydrogen half-reaction with their CBM falling up to 2 eV above the redox potential. Not all of the slabs can kinetically support the catalysis as the band gaps are too narrow to provide the sufficient overpotential. The (100) and (101) surfaces fall into this category with band gaps of 1.69 and 1.21 eV, respectively. The (100) and (1–12) could thermodynamically bring about the oxygen half-reaction; however, as the VBM of surfaces were mere 0.1 eV below the redox potential, the holes would not have sufficient energy to oxidize water. All other surfaces had the VBM well within the redox window, so oxygen evolution on LTA would not be feasible. This agrees well with experimental reports where bare LTA performs poorly in a one-step water-splitting scheme but well when employed in a Z-scheme as the hydrogen evolution photocatalyst.^15,16^

None of the LTC surfaces studied could thermodynamically and kinetically photocatalyze the overall water splitting; however, all but (010) have their VBM below the oxygen evolution half-potential (see Figure 8b). On the (010) surface, CBM was appropriately positioned for hydrogen evolution with sufficient overpotential; however, the VBM sat at −5.63 eV so overall splitting was not achievable. Similarly, the CBM in (110) and (−211) surfaces allowed for effective hydrogen evolution, but the overpotential on the VBM was inadequate to satisfy the kinetic requirement of photocatalysis. The overpotential for oxygen evolution was suitable for (100) and (101) which account for 47% of the total Wulff shape area. In the case of the (100) surface, CBM directly coincided with the hydrogen half-reaction potential, while the band gap of the largest facet was too narrow for the surface to straddle the redox potential with VBM at −5.07 eV.

The (102) surface appeared to be an outlier in both systems and was excluded from discussion so far. The VBM positions seemed in line with other surfaces, but both exhibit minuscule HSE06 calculated band gaps of −0.05 eV for LTA and 0.38 eV for LTC. The band gaps remained constant between PBEsol relaxation and the HSE06 single-shot calculation used to obtain energy values for the band alignment which was most unusual as PBEsol band gaps are systematically underestimated and increase significantly after a hybrid functional is applied.

Surface band gaps calculated for band alignment can aid in the explanation of the apparent overestimation of bulk HSE06 band gaps compared to experimental data. Generally, the surface band gaps were smaller than the calculated bulk band gap for the material. Accounting for the relative sizes of surfaces and their respective estimated band gaps, the surface-averaged band gap lowers to 1.68 eV for LTA and 1.51 eV for LTC, with the (102) surfaces excluded from the calculation. As these band gaps clearly underestimate the experimental bulk band gaps, it is possible a combination of effects can explain the disparity—the surfaces may have influenced the experimental band gaps, but also the computational band gaps may differ as they are calculated in athermal conditions.

Some conclusions based on the combination of Wulff shapes and band alignments can be drawn. The surfaces best suited for hydrogen production were in both materials at the flat ends of columnar particles, while the band gaps of the side facets were too narrow to provide enough driving force for the half-reactions to occur. The efficiency of water splitting could be maximized by focusing synthesis on methods that favor formation of end surfaces, rather than elongating the crystallites in the *b*-direction. Additionally, the band alignments provided here could help with the choice of cocatalysts tested in the future to maximize the potential for water splitting.

However, like with surface energies, the band alignments calculated here assume the surfaces are in a vacuum rather than in aqueous solution. It has been experimentally shown that solvation affects the position of band edges with respect to H^+^/H_2_ potential, so that the band alignment reflects the intrinsic nature of semiconductors.^44^ Because of the electron transfer between water and the semiconductor, a downward shift of band edges is expected for p-type semiconductors, while n-type semiconductors exhibit an upward shift. Considering LTC has been successfully doped with Mg and Sc, indicating n-type behavior, an upward shift of the band edges may be expected.^23,45^

Quantifying the shift by using solvation calculations would be prohibitively expensive for these quinary systems, but some conclusions may be drawn from the literature. In a study comparing experimental work with theoretical models, Stevanović et al. demonstrated that an upward shift of 0.5 eV is reasonable when pH is equal to the point of zero charge, on a number of metal oxides.^44^ Experimental data also show an upward shift for TiO_2_, Ta_2_O_5_ (0.14–0.64 eV), and WO_3_(2 eV) at pH = 1.^44^ Thus, it can be argued that an overall upward shift of anywhere between 0.2 and 0.6 eV could be expected, increasing the overpotential for hydrogen evolution reaction and so making both LTA and LTC good hydrogen-evolution photocatalysts. However, the calculated band gap of the bulk system was overestimated compared to the experimental values, so it is also possible that the valence band is actually slightly lower in energy and as such counterbalancing the environment-driven band alignment shift.

## Conclusion

The electronic structure of bulk LTA and LTC was analyzed by hybrid DFT methods. The bulk band gap and charge carrier effective masses were found to be appropriate for photocatalytic water splitting in LTC, while the electron effective mass was too high for effective hydrogen evolution in LTA. For each material, surfaces up to a maximum Miller index of 2 were cleaved from the bulk and fully relaxed. All calculated surface energies were small, in the range of 0.8–1.5 J m^–2^, and Wulff reconstructions comprised seven of the surfaces for both systems. Good agreement was found between experimentally realized single crystals and the Wulff shapes predicted in this study.

In terms of band alignment, the study confirmed experimentally observed trends. Combining Wulff shapes with band alignments, only 30% of LTC surface area can participate in hydrogen evolution reactions with sufficient overpotential but 72% can catalyze oxygen evolution reaction. LTA on average exhibited larger band gaps which led to appropriate positioning of the conduction band for photocatalytic hydrogen production—the entire surface area of LTA Wulff shape could promote thermodynamic hydrogen evolution. As the valence bands were too high in energy in LTA, neither of the materials were appropriate for one-step overall photocatalytic water splitting. However, with some band structure fine-tuning or choice of appropriate cocatalyst loading both LTA and LTC could be employed in Z-scheme photocatalytic water splitting.

## Supporting Information Available

Structural data of the bulk structures and surface slabs, sample relaxation inputs, all surface energies and energies for band alignments (PDF)
