# Supplementary material for: Understanding the Photocatalytic Activity of La5Ti2AgS5O7 and La5Ti2CuS5O7 for Green Hydrogen Production: Computational Insights
Source: ACS Appl Energy Mater. 2022 Jan 26;5(2):1992–2001. doi: 10.1021/acsaem.1c03534 (PMC8889536; doi:10.1021/acsaem.1c03534)
Supplement: Supplementary file 1 — ae1c03534_si_001.pdf [file ae1c03534_si_001.pdf]

Supporting Information

Understanding the Photocatalytic Activity of $La_5Ti_2AgS_5O_7$ and $La_5Ti_2CuS_5O_7$ for Green Hydrogen Production: Computational Insights

Katarina Brlec,[†,‡] Seán R. Kavanagh,[†,‡,¶] Christopher N. Savory,[†,‡] and David O. Scanlon[*,†,‡]

† Department of Chemistry, University College London, 20 Gordon Street, London WC1H 0AJ, UK
‡ Thomas Young Centre, University College London, Gower Street, London WC1E 6BT, UK
¶ Department of Materials, Imperial College London, Exhibition Road, London SW7 2AZ, UK

E-mail: d.scanlon@ucl.ac.uk

Supporting information contains structural data of bulk and surface slabs, sample relaxation inputs, all surface energies and energies for band alignments. This material is available free of charge via the Internet at https://doi.org/10.5281/zenodo.5110123
